# A Research on Preparation and Application of the Monolithic Catalyst with Interconnecting Pore Structure

**DOI:** 10.1038/s41598-018-35021-2

**Published:** 2018-11-09

**Authors:** Sang-Bing Tsai, Hailing Ma

**Affiliations:** 1 Zhongshan Institute, University of Electronic Science and Technology of China, Guangdong, 528402 China; 2 Research Center for Environment and Sustainable Development of the China Civil Aviation, Civil Aviation University of China, Tianjin, 300300 China; 3 Institute of Mechanics, Chinese Academy of Sciences, Beijing, 100190 China; 4 School of Science, Jinggangshan University, Jian, 343009 China

## Abstract

In recent years, the monolithic material has been developed increasingly in the high performance liquid phase field, and it could also be applied in the field of catalyst, as a monolithic catalyst carrier, since it has a large specific surface area, and could be customized based on the mould. The monolithic catalyst is characterized with many advantages such as low bed pressure, high physical efficiency and small amplification effect. The most impotant part refers to the preparation of copper-based catalyst. The impregnation method is used to produce CuO-ZnO monolithic catalyst and CuO-ZnO-ZrO_2_ monolithic catalyst with the prepared monolithic silica-alumina carrier. The fixed bed microreactor is used to investigate the effect of copper-based catalyst on the process in which carbon dioxide is used to produce methanol through hydrogenation. The metal salt is added into the sol-gel process, which could form the M-O-Si bond, thus make the metal-containing catalytic material obtain good mechanical strength, and make it possible to be introduced into the acidic center generally. The metal-containing catalytic material carrier also has macropores and mesopores. The presence of large pores could make the molecular mass transfer more effective, while the presence of mesopores could increase the specific surface area of the material. In this paper, the experimental study has been conducted on the production of methanol through hydrogenation of CO_2_ under different catalysts, to mainly investigate the effect of catalysts with different catalytic performance on the reaction.

## Introduction

Porous materials have been widely used in oil refining, chemical, environment protection, automobile and other industries for a long time, due to its relatively large specific surface area. In general, the porous materials are classified based on the pore size, i.e. microporous catalyst materials (pore size less than 2 nm), mesoporous catalytic materials (pore size between 2–50 nm), and macroporous catalytic materials (pore size greater than 50 nm). In most catalytic reactions, sizes of the reactant and product molecules are less than 1 nm, so the microporous zeolite molecular sieve has been used commonly as a catalyst and adsorption material. However, it may become powerless in some macromolecule reactions, due to its pore size limitation. In 1992, Mobil reported the MCM-41 mesoporous material that extends the range of porous materials from ordered sub-nanopore to ordered nanopore, which is a milestone progression in the field of porous materials^1^. In recent years, as the orderly mesoporous materials and macroporous materials have been reported successively, research on macroporous materials has become a very active sector in the field of material science^2–6^.

Conventional catalyst carriers are mainly made of aluminum oxide, silica gel, mesoporous molecular sieve and other porous materials. Generally, such materials are in the shape of a small ball, the surface of which is covered with many small pores, with poor interconnectivity in most cases. When the reaction molecules pass through these pores, they may be trapped easily, while the molecules of small volume are more likely to get inside due to the diffusion effect. The molecules that have got inside the pores could get out due to the diffusion effect as well, thus could realize the close contact between the molecules and the activity centers on the catalyst surface. In the common carrier materials, the feed material flows in laminar flow pattern, and the molecules get inside and out of pores due to the diffusion effect. Therefore, if the speed is too fast, there will be no enough time for the molecules to get inside the pores, and the reaction effect will be adversely affected significantly, leading to low reaction efficiency.

As it is the basic mechanism of the sol-gel method, metal compounds like inorganic salts or metal salts cause the hydrolysis and polycondensation reactions in a water-based solution, which could make the solution undergo the process from the sol state to the gel state; and then the gel is dried, baked and processed by other measures, to produce a solid uniformly-distributed material. In 1846, the sol-gel method was reported for the first time by Ebelmen *et al*., who found that the hydrolysis reaction happened when ethanol was mixed with SiCl_4_ in a humid atmosphere, and SiO_2_ was produced. In 1939, Berger reported that the sol-gel reaction method could be used to produce a single oxide film, making such a method particularly concerned in the science community. Since the reaction condition of sol-gel method is mild, and it is implemented under low temperature and normal pressure in most cases, the sol-gel technology has been widely applied in producing films, coatings, glass and other materials^7–9^.

The silica gel material is mainly used as a chromatographic column, which is produced with the sol-gel technology in most cases. The material could be used to produce siloxane oligomer with the sol-gel method, through hydrolysis of TMOS or TESO under the catalysis. In 1991, Nakanish *et al*. reported the process to produce silica gel monolithic material, in which the sol-gel method was used, and the water-soluble organic polymer sodium styrenesulfonate was added into tetramethoxysilane under the action of nitric acid, to produce the silica gel monolithic material with different structures; and they also investigated the effect of different conditions on the silica gel material. Then they replaced the organic polymer with polyacrylic acid, polyethylene oxide and other substances, to produce the silica gel material, and also investigated the effect of different conditions on the material^10–14^. Wang Yue produced the silica gel monolithic column of TMOS-PEG system with TMOS and PEG as the material, and defined the optimum reaction parameters by means of orthogonal test. He increased the hardness of the material through changing the aging procedure, and investigated the effect of soaking treatment with ammonia water of different concentrations on the pore size^15–18^. The material is mainly applied in the chromatography field, and the application of such a material in the catalyst field has not been reported in any literature currently.

A solid catalyst is usually composed of three parts, i.e. active component, catalyst promoter and carrier, but some catalysts are only composed of two parts, i.e. active component and carrier. The initial purpose of the carrier is only to disperse the active component or to improve the abrasive resistance and impact strength of the catalyst. But it is found in later applications that the carrier of different materials will make the performance of catalyst vary significantly, so more and more attention has been paid to the development and selection of the carrier. A carrier should not only have sufficient mechanical strength and a specific surface with pore structure, but also have a shape suitable for the reaction. So the development of monolithic catalyst is increasingly fast. The monolithic carrier is mainly distinguished in features such as heat transfer, mass transfer and pressure drop, compared with the traditional granular carrier catalyst. The heat transfer effect of the conventional cellular monolithic catalyst is not ideal, because its individual channels are independent of each other and the radial heat transfer effect is poor during the reaction, leading to an adverse impact on the performance of the catalyst due to the exothermic and endothermic effect of reaction^19–26^.

## Preparation of Catalyst

### Mechanism for Preparation of Silica Gel

The silica gel monolithic material is prepared through hydrolysis and polycondensation of the silicon source. The polycondensation product of the silicon source then grafts with the added polymer to form a homogeneous mixed solution system, which undergoes a shift from sol to gel and the phase separation. During the process of separation, since slight changes occur in the mixed solution system, the system further undergoes a shift from gel to solid, which could be divided into three stages, i.e. diffusion, liquid flow and formation of similar framework. Finally, a three-dimension morphological structure is obtained, and a network structure is formed gradually. After the inter-framework solvent has been removed, macropores of micron order in size are obtained, while mesopores are formed due to the aging and the exchange of solvent. Therefore, the porous silica gel monolithic material with multiple pore channels is produced.

### Mechanism for Sol-gel Reaction of Alkoxy Silane

The mechanism for sol-gel transformation of alkoxy silane is composed of two reactions, i.e. hydrolysis and polymerization. The dynamic Flory-Stockmayer theory in the sol-gel transformation is also called as the “dendritic polymerization” theory, which indicates that in the typical sol-gel transformation model, the dendritic cluster does not form a closed loop in the reaction; the volume of dendritic cluster increases with the increase of average degree of polymerization; and finally the tree cluster forms on the gel point, and spreads out indefinitely. The remaining small clusters are left in the sol, adsorbed on the framework, and it makes their dispersion reduced. The bicontinuous structure formed by the metastable decomposition enlarges as the time passes. And after the formation of the tree cluster, the gel process starts, and then the activity of framework structure is limited. Which process will take precedence, phase separation or sol-gel transformation? It is a question that needs to be considered.

**Figure 1:**
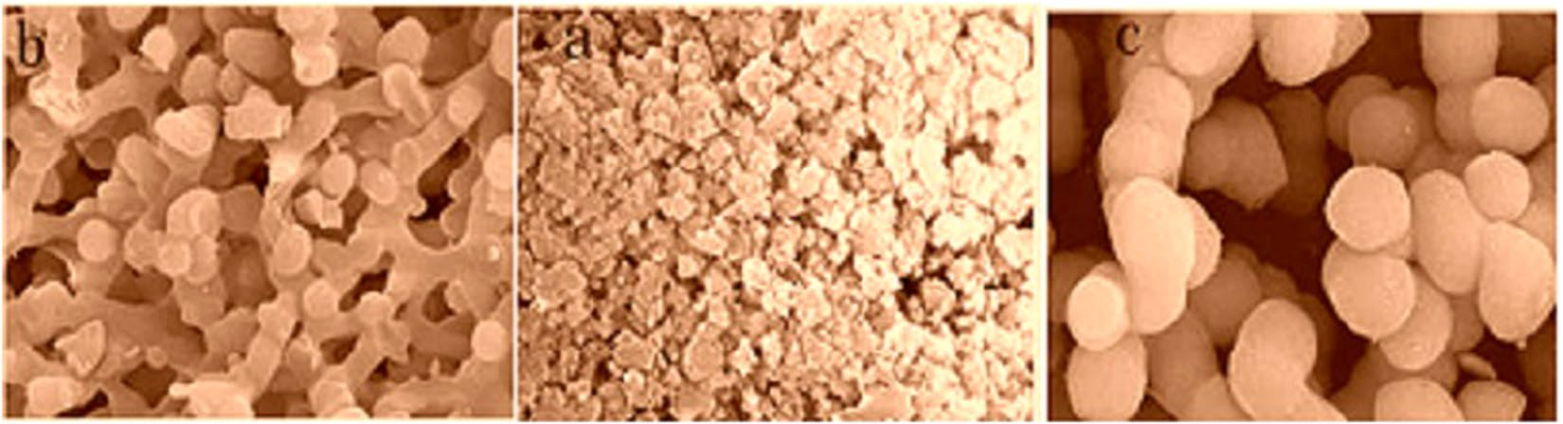
Different structure of silica gel monolithic materials caused by different separation and gelation rate.

Figure 1 shows three types of internal appearance: picture *a* refers to the silica gel monolithic material with a porous structure of nanometer order; picture *b* refers to the silica gel monolithic material with a double-pore structure; and picture *c* refers to the silica gel monolithic material with a granular-stacked structure. The difference in internal appearance among above three types of material is determined by the duration of phase separation and gel transformation. The silica gel material with a porous structure of nanometer order will be obtained, when the rate of gel process is faster than that of the phase separation process; the silica gel material with a large-granule stacked structure will be obtained, when the rate of phase separation process is faster than that of the gel process,; and the silica gel monolithic material with a double-pore structure will be obtained, only when the rate of gel process is consistent with that of the phase separation process.

### Modification of Porous Catalyst Material by Metal and Metal Oxide

It could be considered as a very effective way to use metal and metal oxide to improve the catalytic performance of a catalyst. The introduction of metal and metal oxide could change the catalytic activity center of the acid-base catalyst or the redox catalyst, could improve the anti-water vapor performance of the catalyst, resulting in a better durability, or could increase the hardness of the catalyst material and improve the stability of the framework. Therefore, it is critical for such a kind of catalyst material to study the selection and introduction of metal and metal oxide, their state in the material or the position of active center. Takahashi *et al*. have produced the silica gel monolithic material with a double-pore structure with TEOS and PEO, and have added aluminum nitrate therein, which contributes to the formation of numerous Brønsted acid sites in the material, leading to a good catalytic cracking activity of the material.

## Preparation of Copper-based Catalyst

### Preparation Method of Copper-based Catalyst

Currently, the copper-based catalyst has been widely used in most of studies on production of methanol through hydrogenation of carbon dioxide, the main active component system of which includes CuO/ZnO, CuO/ZnO/Al_2_O_3_, CuO/ZnO/ZrO_2_, CuO/ZnO/ SiO_2_, CuO/NiO/TiO_2_, CuO/ZnO/SiO_2_ (ZrO_2_)-MxO_Y_ (M = Li, Na, K, Cs, Ca, La, Y, Ce, Al, Mn, Zr, Th, Si, Ti, Mg and Ba etc.). The preparation method of copper-based catalyst mainly includes co-precipitation method and impregnation method.

#### Co-precipitation Method

The co-precipitation method is a method in which a precipitating agent is added to a mixed solution of copper salt and other metal salts to precipitate two or more kinds of cations contained in the solution to produce the precursor of precipitated mixture or solid solution, which then is used to produce the composite oxide through filtration, washing and thermal decomposition. However, addition of the precipitating agent may cause the local concentration to be too high, resulting in agglomeration or uneven composition. In a study performed by Liu Zhijian and Liao Jianjun *et al*., the co-precipitation method has been adopted to produce CuO-ZnO, and CeO_2_ has been used to modify it through impregnation. After the modification has been made, the crystal particle size of CuO enlarges significantly, and the adsorption volume of carbon dioxide decreases, but the activity of carbon dioxide has been improved in production of methanol through hydrogenation. ZHAO Yunpeng *et al*. have developed a copper-based catalyst (CuO-ZnO-TiO_2_) for hydrogenation of carbon dioxide by means of the parallel flow precipitation method, and as it is shown in a series of experiments, after the surfactant is added, the reduction temperature of the catalyst decreases, which could make CuO in the catalyst be reduced to the metal copper sufficiently, thus could guarantee the catalytic activity of catalyst. Xu Zheng and Qian Zaihu *et al*. from the Department of Chemistry, Jilin University, have carried out a study to investigate the preparation process of copper-based catalytic system with co-precipitation method, as well as the mechanism of reaction in which the methanol is produced through hydrogenation of carbon dioxide under the catalytic action of CuO-ZnO-ZrO_2_, and the active reaction center on the surface. The result shows that the valence state of copper in the active center is Cu+ and CuO. Xu Yong and Wang Ren from the Industrial Catalysis Institute, East China University of Science and Technology, have conducted a study on the catalyst Cu-ZnO-Al_2_O_3_/Cu0-ZnO-ZrO_2_ produced with the co-precipitation method and the reaction characteristics of the catalyst, and the result shows that the conversion rate of carbon dioxide reaches 38.6%, and the single-pass yield of methanol is 16.74%, when the proportion of Cu, Zn and Zr is 45: 45: 10, under a condition listed as follow: 240 °C, 3500h_1 and 2Mpa.

#### Impregnation Method

The impregnation method is a method in which the copper salt solution is impregnated onto the carrier and then subjected to drying, baking and other processing procedures to produce a copper-based catalyst. Zhong Shunhe *et al*. have produced a bimetallic Cu-Ni catalyst carried by ZrO_2_-SiO_2_, through the equivalent-volume impregnation method. Since the composite carrier has a large specific surface area, it could improve the performance of a catalyst. Therefore, the selectivity of methanol could reach 90% or more in the reaction under a pressure of 0.5 Mpa and at 170 °C. Xie Hongjuan *et al*. have produced CuO/SiO_2_ catalyst by the impregnation method, with water, ethanol and acetone taken as the dispersant. They have concluded that the yield of methanol is highest when ethanol and acetone are taken as the dispersant. Wen Lidan and Li Jinlai have produced a Cn/Zn/TiO_2_-carried catalyst by the impregnation method, and have investigated how the contents of Zn and Cu may affect the reaction performance of the catalyst.

### Effect of Promoter Added on Copper-based Catalyst

Currently, the CuO-ZnO catalyst is the most common copper-based catalyst used in production of methanol through hydrogenation of carbon dioxide, which has good effect but poor stability. Introduction of a promoter into the copper-based catalyst could improve the catalytic effect significantly. In most cases, a metal oxide is taken as the promoter. Introduction of such an additive could increase the specific surface area of the catalyst, and increase the number of active centers; in addition, some metals and their oxides could also increase the activity of active center. Promoters under research currently include Zr, Ce, Mn, La, Ag, K and Pt etc.

Huang Shupeng *et al*. have produced CnO-ZnO-Al_2_O_3_ catalyst by the co-precipitation method, modified with 6 types of metal oxides as adjuvant. The result of experiment shows that addition of ZrO_2_ and Ag_2_0 has increased the conversion of CO_2_ and the selectivity of methanol under certain conditions; addition of ZrO_2_ has increased the specific surface area of the catalyst; and addition of Ag_2_0 has brought new active center, Ag+, for the catalyst.

Wu Ying and Wu Binfu *et al*. have produced Cu-Zn-Al-Mn catalyst by the co-precipitation method, and as it is shown in the evaluation on the activity of catalyst, addition of Mn could increase the activity of the catalyst and the conversion rate of CO_2_. Based on the XRD test of the catalyst, it is found that addition of Mn could inhibit the growth of copper oxide crystal grains, leading to a better distribution of copper oxide grains in the catalyst, thus could increase the reactivity of the catalyst.

Lin Guiming and Shan Yongkui *et al*. have conducted a research to investigate the effect of manganese and lanthanum introduced into the catalyst on the activity of the catalyst by introducing Mn and La into CuO / ZrO_2_ copper-based catalyst. The result of research shows that addition of Mn could disperse the active component, and addition of La could inhibit the crystallization of ZrO2 particles. In addition, it is also concluded that Mn and La could form a synergistic effect.

Liu Yuan and Qin Yongning *et al*. have investigated the effect of potassium promoter on Cu0/ZrO_2_ copper-based catalyst. It could be found in the test that potassium exhibits an electronic effect and could be used as an electron promoter. Addition of potassium has achieved the content adjustment of carbon dioxide in the reaction, and ultimately has increased the conversion rate of carbon dioxide in the reaction.

### Active Center for Production of Methanol through Hydrogenation of Carbon Dioxide

The active center of the copper-based catalyst, used in production of methanol through hydrogenation of carbon dioxide, has been investigated by researchers in a number of discussion and study activities, and it has been divided into three types as below. (1) As it is believed by many researchers, the synergistic effect between Cu+ and the adjuvant will form the active sites in synthesis of methanol; the copper could be dissolved by the matrix surface of these solid solvents; and the active sites derives from the synergistic effect between copper and solvents. Li Jitao and Chen Mingdan *et al*. have conducted a research to investigate the effect of ZnO on the copper-based catalyst for methanol synthesis, and the result shows that the more ZnO is contained in the catalyst, the more intermediates (CH_X_ or CH_x_O) are adsorbed on the surface of the catalyst; and as the concentration of Cu+ increases on the surface of catalyst, the catalytic effect of catalyst improves. It suggests that the synergistic effect between Cu+ and ZnO increases the activity of the catalyst. (2) Some researchers believe that C/ is the unique active site. The methanol production reaction happens only on the surface of C/, and ZnO or Al_2_O_3_ just plays a role to disperse the active compositions, or to prevent the copper particles from sintering. (3) Some researchers believe that two-dimension C^-C/ is the active center, and the ratio of Cu+/C/ determines the features of catalyst, such as the thermal stability. Liu Yanxia *et al*. have produced a series of isolating agents to control the ratio of C_U_+/C/. It is found that addition of y-A1_2_O_3_ could achieve the optimum control on the ratio of Cu+/CuO. Cao Yong *et al*. have produced CuO-ZnO-Al_2_O_3_ catalyst by co-precipitation method. They have analyzed the relative proportions of Ci+ and C/ species on the surface of the catalyst with XAES. It is considered that the synergistic effect between Cn+ and C/ increases the catalytic activity of the catalyst.

### Reaction Mechanism for Production of Methanol through Hydrogenation of Carbon Dioxide

Currently, two opinions are supported with regard to the reaction mechanism for production of methanol through hydrogenation of carbon dioxide. First, carbon dioxide converts to carbon monoxide prior to involvement in the production of methanol through hydrogenation. Second, carbon dioxide is directly involved in the production of methanol through hydrogenation without conversion. As the study goes further, the second opinion, i.e. direct involvement of carbon dioxide in the production of methanol, has got more and more support. Xu Zheng *et al*. have investigated the reaction mechanism for production of methanol through hydrogenation of CO_2_ under the catalytic action of CuO-ZnO-ZrO_2_ catalyst by using TPSR and other technical routes. And the result verifies that methanol is directly produced by CO_2_ and H_2_, without conversion into CO, and the intermediate product is formate.

## Method

Firstly, the conditions for preparation of silicon-aluminum monolithic material and monolithic catalyst have been discussed respectively. The scanning electron microscopy is used to observe the microstructure of the catalyst carrier. The AS-1C-TCD type physical adsorption instrument is used to determine the specific surface area, mesopore size distribution and adsorption isotherm of the catalyst carrier. FT-IRNicolet6700 Fourier Infrared Spectrometer is used to determine the framework of sample, and to characterize the functional group. The Synchronous Thermal Analyzer of STA449F3 Model is used for TG-DSC analysis. The D8-FOCUS X-ray Diffractometer is used to observe the microstructure of the monolithic material and catalyst. And the activity of catalysts has been evaluated respectively.

### Instruments and Reagents

Main reagents and apparatuses used in the experiment are shown in Tables 1 and 2 respectively.

**Table 1:** Experimental reagents.

Name	Purity	Source
Copper nitrate	Analytical reagent (AR)	Tianjin Kemiou Chemical Reagent Co., Ltd.
Ammonia	Analytical reagent (AR)	Tianjin Kaitong Chemical Reagent Co., Ltd.
Acetic acid	Analytical reagent (AR)	Tianjin Kemiou Chemical Reagent Co., Ltd.
Ethyl orthosilicate	Analytical reagent (AR)	Tianjin Fuchen Chemical Reagent Factory
Nitric acid	Analytical reagent (ARmw: 10000)	Sinopharm Chemical Reagent Co., Ltd.
Polyethylene glycol	Analytical reagent (AR)	Tianjin Dongli Tianda Chemical Reagent Factory
Aluminum nitrate	Analytical reagent (AR)	Tianjin Kemiou Chemical Reagent Co., Ltd.
Zinc nitrate	Analytical reagent (AR)	Tianjin Kaitong Chemical Reagent Co., Ltd.
Ethanol	Analytical reagent (AR)	Tianjin Kaitong Chemical Reagent Co., Ltd.
Zirconium nitrate	Analytical reagent (AR)	Tianjin Kemiou Chemical Reagent Co., Ltd.

**Table 2:** Experimental apparatus.

Name	Model	Manufacturer
Electronic balance	BS124S	Beijing Sartorius Instrumentation System Co., Ltd.
Super constant temperature water bath	CS501	Chongqing Experimental Equipments Factory
Electric blast drying chamber	101—0	Huanghua Aerospace Instrumentation Factory
Muffle furnace	MFL-2000	North China Experimental Instrumentation Co., Ltd.
Six-connection magnetic heating mixer	HJ-6	Jintan Huafeng Instrumentation Co., Ltd.
Micro-reactor chromatography experimental device	CN-WF02	Tianda Beiyang Chemical Equipment Co., Ltd.
Circulating water vacuum pump	SHZ—IIIB	Zhejiang Linhai Precision Vacuum Equipments Factory
Gas phase chromatographic instrument	GC9800	Shanghai Science and Technology Innovation Center
Quartz automatic double pure water distiller	1810-B	Jintan Huafeng Instrumentation Co., Ltd.
High purity hydrogen generator	CY500-II	Beijing Keep-Science Analysis Sci&Tech Co., Ltd.

### Experiment Method

#### Preparation of Silicon-Aluminum Monolithic Catalyst Material

Weigh out deionized water 9.5 mL, concentrated HNO_3_ (65–68%) 1.06 mL, and add them into water; weigh out polyethylene glycol (PEG) 1.1500 g, aluminum nitrate (Al (N_3_) 3.9H_2_O) 2.2100 g, and add them successively. Stir the mixture at the appropriate speed until the solution is uniformly dissolved, and then weigh out the ethyl silicate (TEOS) and added it thereto. Stir the mixture rapidly for 30 minutes. Put the mixture into a mold tube (® = 8 mm), seal the tube, and put it into the constant-temperature water bath to warm it at 45 °C for 24 h; take the wet column out of the tube, and allow it to dry naturally at room temperature. Dry it at room temperature for 72 h. Bake the dried column at 600 °C for 3 h. After baking, the monolithic silicon-aluminum material is produced and ready for further use.

#### Preparation of Monolithic Catalyst

Prepare copper nitrate and zinc nitrate solution of 1 mol/L respectively, and prepare the impregnation solution according to a certain proportion of the two solutions. Pour the impregnation solution into a sealable glass bottle. Put the prepared monolithic catalyst carrier in the impregnation solution and immersed in the constant-temperature water bath at 50 °C for 24 h. Take the carrier out and put it in a watch glass for drying. Increase the temperature quickly to 120 °C, and keep the temperature for 8 h; then burn it at 550 °C for 4 h. The carrier of CuO-ZnO catalyst is then produced. CuO-ZnO-ZrO_2_ monolithic catalyst could also be prepared with the same method.

## Analysis and Discussion of Experimental Results

### Effect of Different Proportion on Catalyst

The performance of industrial catalysts depends largely on their chemical composition and physical structure. As the preparation proportion and method are different, although the compositions and operating conditions are identical, the performance of the catalysts produced may still be very different. In this experiment, the effects of the proportion on the structure and catalytic performance of catalyst has been investigated with different preparation conditions.

#### Determination on Activity of Catalysts with Different Preparation Proportions

**Figure 2:**
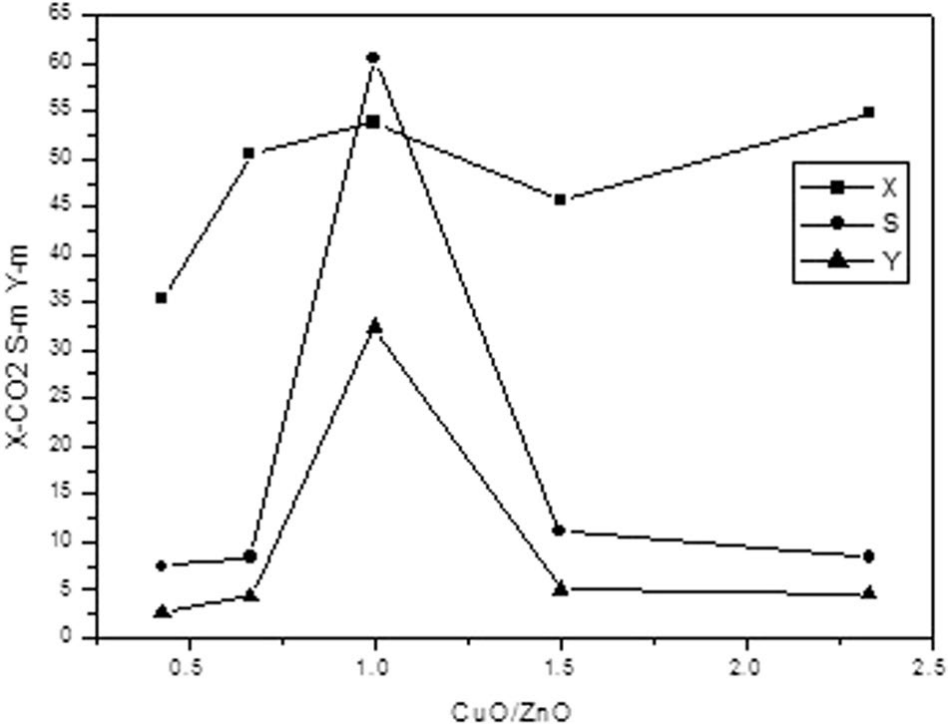
Change in Reactivity of Catalyst.

As it is shown in Fig. 2, the reactivity of a single catalyst, under the same reaction conditions, may be impacted significantly by the different preparation proportions, and it could be analyzed as follows:

In this experiment, it can be seen from the data in the figure that only when the CuO/ZnO ratio is 1:1, the catalyst may have the strongest activity, and the conversion of carbon dioxide, the selectivity of methanol and the yield of methanol all reach the peak point. In the event of any other proportion, they may show an increasing trend as the proportion of CuO increases, and show a decreasing trend as the proportion of CuO decreases.

#### Determination of Specific Surface Area of Catalysts under Different Preparation Proportions (Table)

As it is shown in Table 3, the specific surface area of a single catalyst, under the same reaction conditions, may be impacted significantly by the different preparation proportions, and it could be analyzed as follows:

**Table 3:** Relationship between Composition and Specific Surface Area of Catalyst.

Composition of Catalyst	Specific Surface Area/m^2^ g^−1^
70 CuO 30 ZnO	40.0702
60 CuO 40 ZnO	48.6745
50 CuO 50 ZnO	60.0702
40 CuO 60 ZnO	46.3009
30 CuO 70 ZnO	39.3443

In this experiment, it can be seen from the data in the table that only when the CuO/ZnO ratio is 1: 1, the catalyst may have the largest specific surface area. In the event of any other proportion, it may show an increasing trend as the proportion of CuO increases, and show a decreasing trend as the proportion of CuO decreases.

### Effect of Carrier on the Catalyst

The carrier is a special component of the solid catalyst. It could increase the surface area, improve the heat resistance and mechanical strength of the catalyst, functions as an anti-toxic agent or reduce the generation of by-products, and withstand various effects caused by temperature change, phase change and other reasons. The presence of a carrier could always determine the macro-physical structure of the catalyst. Different carriers have a significantly different effect on the activity of the catalyst. The initial use of the carrier is intended only to increase the specific surface area of the catalytic active substance. Otherwise, it seems to be an inert substance. However, it is found later that the role of the carrier is complex. It often reacts with the catalytic active substance to alter its chemical composition and structure, thus to change the performance of the catalyst, such as activity and selectivity. Therefore, sufficient attention shall be paid to the selection and preparation of the carrier.

1. Alumina is the commonest carrier used in industrial catalysts, due to its low cost, high heat resistance, good affinity to active component, and weak acidity.
2. The chemical composition of silica gel is SiO_2_, usually produced through water glass acidification.
3. The main component of activated carbon is C, with trace amount of H, O, N, S and ash etc. Although the amount of these substances is small, they have a certain impact on the nature of activated carbon. Activated carbon has an irregular graphite structure, with various functional groups attached on the surface, such as carbonyl, quinone and carboxyl groups. Activated carbon is characterized with a well-developed porous structure, large surface area and good thermal stability.
4. The molecular sieve has the micropores of the size corresponding to the level of the reaction.

#### Preparation of Catalyst with Different Carriers

In this experiment, two different catalysts have been prepared by the precipitation method: Cu.Zn.Al (molecular sieve);

Firstly, mix copper nitrate, zinc nitrate and aluminum nitrate in accordance with the specified proportion to prepare a solution, and then prepare a certain amount of sodium carbonate solution; add the molecular sieve to the nitrate solution and stir the mixture, and add the sodium carbonate solution until the pH value of the solution reaches 8. Keep stirring for 1 hour, and then allow it to stand for 3 hours. Then filter and dry the product from standing step, and bake it at 343 °C for 2 hours. Cool it down and process it into tablets. The preparation of catalyst is completed.

#### Effect of Carrier on the Activity of Catalyst

Table 4 shows the effect of different carriers on the catalytic performance of the catalyst in this experiment;

**Table 4:** Effect of Carrier on the Catalytic Performance of Catalyst.

Catalyst	Carrier	Conversion Rate of Carbon Dioxide	Selectivity of Methanol	Yield of Methanol
Cu.Zn.Al	SiO_2_	0.0595	0.9617	0.0572
Cu.Zn.Al	r- Al_2_O_3_	0.2030	0.8541	0.1734

As it is shown in Tables 1, 2, 3 and 4, different carriers may have a significantly different impact on the catalytic performance of the catalyst, and it could be analyzed as follows:

The same catalyst, i.e. Cu.Zn.Al, has been used to compare the effect of different carriers in this experiment. The result shows that the catalyst with r- Al_2_O_3_ as the carrier is generally superior to the catalyst with SiO_2_ as the carrier, with regard to the activity of the catalyst, conversion rate of carbon dioxide and yield of methanol, but inferior with regard to the selectivity of carbon dioxide.

### Effect of Reaction Conditions on the Performance of Catalyst

#### Effect of Temperature on the Performance of Catalyst

The effect of temperature on the catalyst performance is shown, and the reaction condition is listed as follow: pressure, 3.0 MPa; H_2_/CO_2_ mole ratio, 4.0; and air flow rate, 2500 h^−1^. It could be seen that the conversion of CO2 increases as the temperature increases, and the selectivity of DME and methanol and the yield of DEM have experienced a process of first increasing and then decreasing, during which these values have reached the peak level at 240 °C, i.e. 25.0%, 11.1% and 4.2% respectively; the selectivity of CO has experienced a process of first decreasing and then gradually increasing, during which the amount CO generated increases significantly at 280 °C, and the selectivity reaches 82.3% at this temperature. Methanol and DME generation reactions are all exothermic reactions, and the CO generation reaction is an endothermic reaction. When the temperature is low, it is not good for the activation of CO_2_, so the conversion of CO_2_ is low. However, when the temperature is high, it is not good for the production of methanol through hydrogenation of CO_2_, but good for reverse water vapor conversion reaction, resulting in decreased selectivity of oxygen-containing organic compounds.

#### Effect of Pressure on the Performance of Catalyst

**Figure 3:**
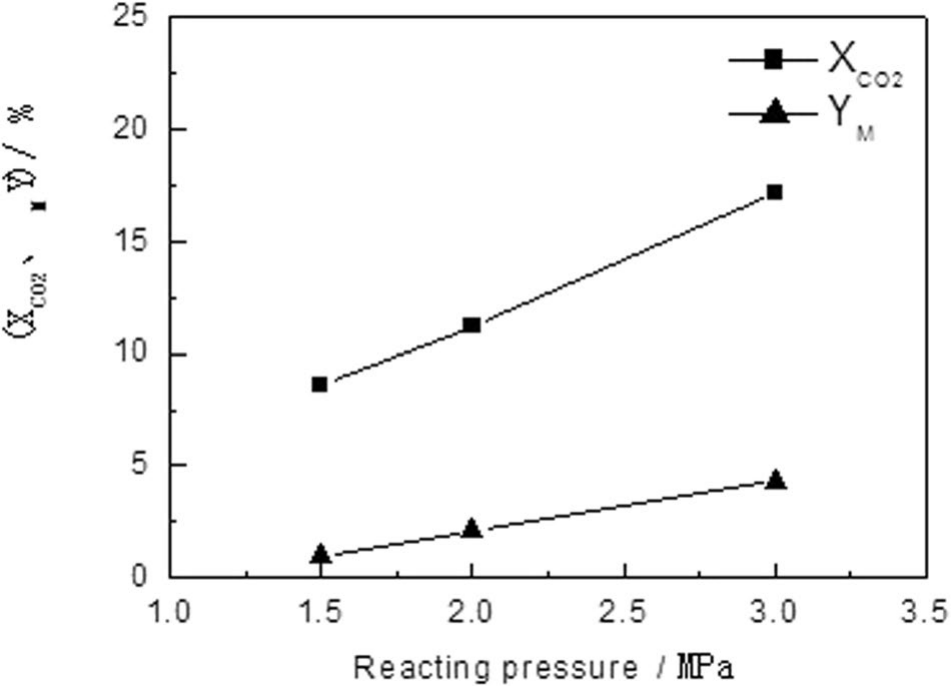
Effect of Reaction Pressure on the Activity of Catalyst.

The effect of pressure on the catalyst performance is shown in Fig. 3, and the reaction condition is listed as follow: temperature, 240 °C; H_2_/CO_2_ mole ratio, 4.0; and air flow rate, 2500 h^−1^. It could be seen that the pressure has a significant effect on the reaction. When the reaction pressure increases from 1.5 MPa to 3.0 MPa, the conversion of CO_2_ is greatly improved, and the selectivity and yield of methanol are also increased significantly. The effect of pressure on the reaction could be explained mainly with two reasons: (1) the increase of pressure is equivalent to the increase of reactant concentration, thus it could make the reaction rate increased; and (2) the reaction for production of methanol through hydrogenation of CO_2_ and CO is a reaction in which the number of moles decreases, in which the increase of pressure is good for movement of the balance towards the positive reaction direction, thus for increase of the CO_2_ conversion rate and the methanol yield. The inverse water vapor conversion reaction is equal-molar reaction, and it could not be affected by the pressure significantly. Therefore, increasing the pressure helps to improve CO_2_ conversion, DME selectivity and yield.

#### Effect of Air Flow Rate on the Performance of Catalyst

**Figure 4:**
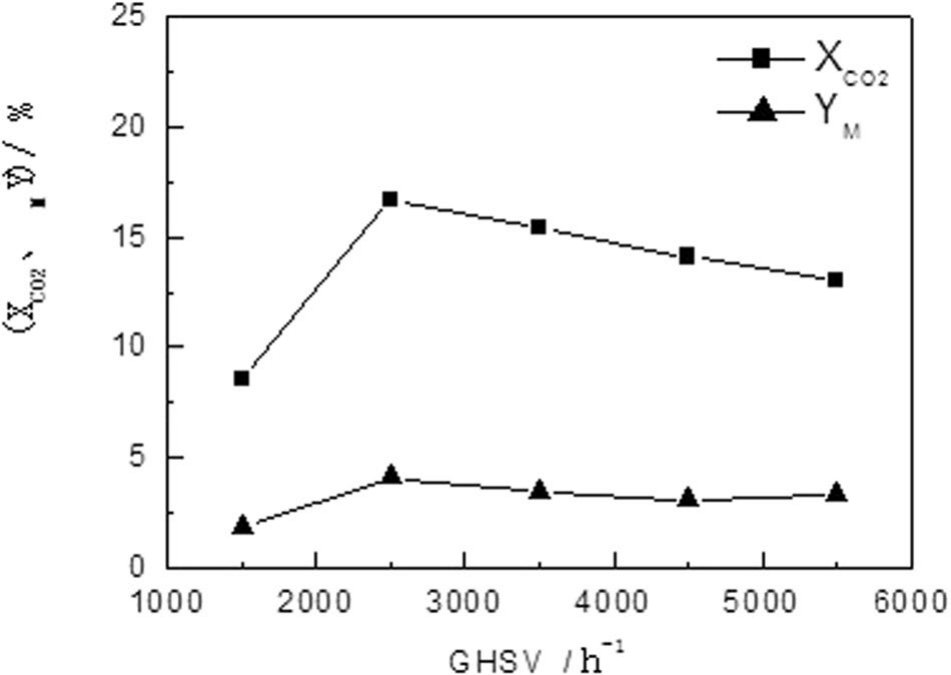
Effect of Air Flow Rate on the Activity of Catalyst.

The effect of air flow rate on the catalyst performance is shown in Fig. 4, and the reaction condition is listed as follow: temperature, 240 °C; pressure, 3.0 Mpa; and H_2_/CO_2_ mole ratio, 4.0. It could be seen that as the air flow rate increases, the conversion of CO_2_ gradually decreases, because the reduced residence time is not enough for part of CO_2_ to react before it leave the catalyst bed; and the selectivity and yield of DME increase first and then decrease as the air flow rate increases, and it is mainly because the DME synthesis reaction is an exothermic reaction, and the increase of air flow rate is beneficial to dissipation of heat. Based on the result of experiment, it is allowed to increase the air flow rate properly in the reaction, with the premise that the conversion should be guaranteed.

#### Effect of H_2_/CO_2_ Ratio on the Performance of Catalyst

**Figure 5:**
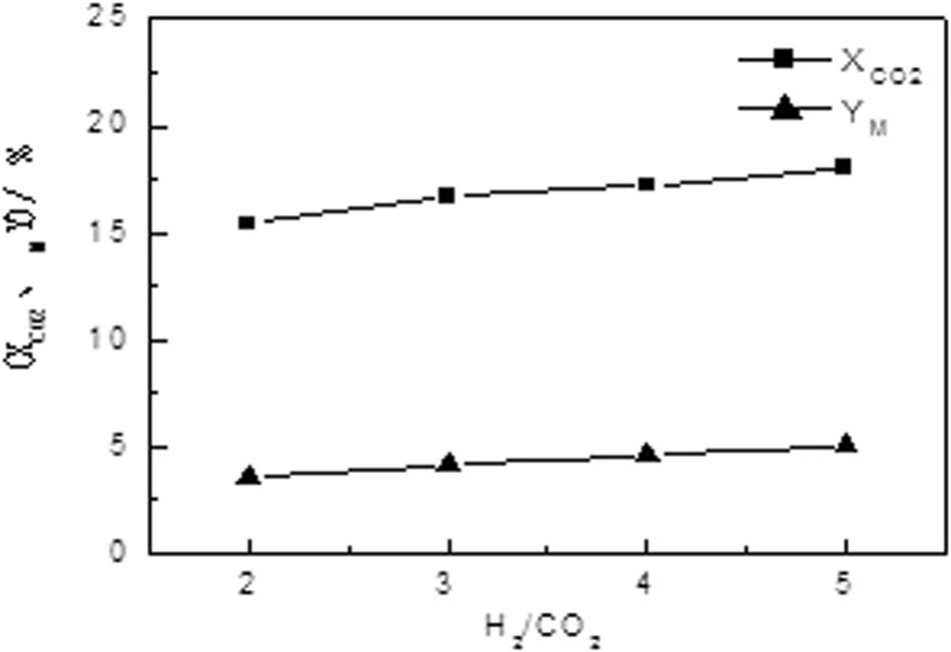
Effect of H_2_/CO_2_ Ratio on the Activity of Catalyst.

The effect of H_2_/CO_2_ ratio (mole ratio) on the catalyst performance is shown in Fig. 5, and the reaction condition is listed as follow: temperature, 240 °C; pressure, 3.0 Mpa; and air flow rate, 1500 h^−1^. It could be seen that increasing the mole ratio of hydrogen to carbon is beneficial to improving the CO_2_ conversion and methanol selectivity, and reducing the CO selectivity. At the same time, it is also observed that the concentration of CO_2_ in the feed gas decreases as the mole ratio of hydrogen and carbon increases, thus the production intensity of the catalyst decreases.

## Conclusion

Under the same reaction condition, CuO-ZnO-ZrO_2_ catalyst is superior to CuO-ZnO catalyst with regard to the reactivity, suggesting that addition of promoter ZrO_2_ could improve the catalytic activity of the catalyst. The effect of reaction temperature on the catalyst is that the catalytic activity of the catalyst is improved with the increase of the reaction temperature, indicated by the increase in both the conversion rate of CO_2_ and the yield of methanol.
